# Models of traumatic brain injury-highlights and drawbacks

**DOI:** 10.3389/fneur.2023.1151660

**Published:** 2023-06-15

**Authors:** Qinghui Zhao, Jianhua Zhang, Huige Li, Hongru Li, Fei Xie

**Affiliations:** ^1^Institute of Physical Culture, Huanghuai University, Zhumadian, China; ^2^Zhumadian Central Hospital, Zhumadian, China; ^3^Faculty of Environment and Life, Beijing University of Technology, Beijing, China

**Keywords:** traumatic brain injury, animal models, cell models, neuroprotective strategies, advantages and disadvantages

## Abstract

Traumatic brain injury (TBI) is the leading cause for high morbidity and mortality rates in young adults, survivors may suffer from long-term physical, cognitive, and/or psychological disorders. Establishing better models of TBI would further our understanding of the pathophysiology of TBI and develop new potential treatments. A multitude of animal TBI models have been used to replicate the various aspects of human TBI. Although numerous experimental neuroprotective strategies were identified to be effective in animal models, a majority of strategies have failed in phase II or phase III clinical trials. This failure in clinical translation highlights the necessity of revisiting the current status of animal models of TBI and therapeutic strategies. In this review, we elucidate approaches for the generation of animal models and cell models of TBI and summarize their strengths and limitations with the aim of exploring clinically meaningful neuroprotective strategies.

## 1. Introduction

TBI is a serious problem worldwide. Approximately 10 million people worldwide suffer TBI each year, and a significant number of patients die as a result, or become temporarily or permanently disabled (1, 2). For example, in 2013, there were 2.5 million emergency hospital visits, 280,000 hospitalizations, and 56,000 deaths from TBI in the United States (3); 2.1 million patients, in Europe, were admitted to hospital due to TBI, 82,000 died (4). It was estimated that the death rate of TBI in China was about 13 cases per 100,000 people, similar to reported death rates in other countries (5). In addition, TBI has been confirmed to be closely associated with epilepsy, Alzheimer’s disease, Parkinson’s disease, chronic neuroinflammation and other diseases (2, 6–10). In order to find a rational treatment plan for TBI, a large number of *in vitro*, cellular and *in vivo*, animal models have been established to study the pathogenesis of TBI. Different animal models have been established to replicate different types of TBI. Although larger animals are more similar to humans in size and physiology, rodents have been widely used in TBI models because of their small size, low cost and easy quantification (10, 11). Early TBI models mainly simulated the biomechanical changes of brain injury, while the models created in recent years have been used to study molecular mechanisms and molecular cascades triggered by head trauma (11–13). *In vitro* TBI models are also potentially important tools to study its pathophysiology. Compared with *in vivo* models, *in vitro* models have the advantages of good repeatability, good controllability, lower experimental costs and fewer ethical problems. Therefore *in vitro* models allow us for large scale production, enabling for reliable and efficient drug screening. Common TBI models can be divided, according to the different modes of injury, into mechanical injury, pressure injury, explosion injury and repetitive minor injury models. In this paper, we review, the above commonly used models, aimed at discovery of effective clinical neuroprotective treatments.

## 2. Mechanical force injury models

### 2.1. Mechanical force-induced TBI animal models

Mechanical force-induced TBI animal models commonly involve weight-drop (WD) and controlled cortical impact (CCI) models.

WD is a commonly used method causing brain damage by impact to the dura mater or skull with falling freely weights. A catheter is used to guide freely falling weights with damage controlled by the weight and height of fall (11, 13–15). The Feeney WD model involves mild, moderate and severe brain injury, simulating concussion and brain contusion, by direct impact to the dura mater causing cerebral cortical injury, with adjustment of the weight hitting the head and the height of free fall (16–21). However, craniotomy can lead to cortical contusion, hemorrhagic lesions, blood-brain barrier damage, immune cell infiltration, and glial cell activation, and prolong modeling time. Shapira Y and Flierl MA et al. (22,23) introduced the closed head WD rodent model by fixing the head on a hard platform to avoid the impact of accelerated diffusion damage on rats and mice later. Weight falls on the unprotected skull to replicate mixed focal/diffuse injury. Due to its simple installation, low cost, and the absence of craniotomy surgery, each modeling time can be controlled within 1 min without any other damage caused by craniotomy surgery, so it has been widely used. However, its drawbacks are the poor accuracy and repeatability of the injury site and the higher mortality rate.

The Marmarou WD model makes two improvements on the Feeney model: (1) Anesthetized rats were fixed to a sponge platform, ensuring instantaneous external force, but avoiding a second impact by pulling out the sponge platform after the blow. (2) A metal sheet with a diameter of 1 cm and a thickness of 0.3 cm is placed to ensure the dispersion of external force, simulating diffuse brain injury. The advantages of this model are simplicity and easy to control conditions. Its disadvantage is a higher fatality rate. Marmarou WD mainly replicates diffuse axonal injury caused by external violence injury such as high altitude fall injury and traffic accident, and Marmarou WD causes extensive and bilateral damage to neurons, axons, dendrites and microvessels, especially in the corpus callosum, internal capsule, optic nerve bundle and other parts. It may also lead to motor and cognitive deficits, such as difficulty walking and memory, which are related to the severity of the injury (10, 14, 15, 21, 24).

In the CCI model, impact force is generated by high-speed moving air driving metal to hit the exposed dura mater directly, causing a degree of brain damage. The CCI WD model is mainly used to replicate clinical situations such as cortical tissue loss, acute subdural hematoma, axonal injury, brain concussion, blood–brain barrier dysfunction and even coma after TBI. The investigators performed a comprehensive neuropathological assessment of TBI models of CCI, indicating that the associated brain injuries may be quite extensive, including acute cortical injury, hippocampal and thalamic degeneration, neuronal loss, vascular rupture, edema, and macrophage accumulation. The degree of injury was controlled by adjusting the duration, speed, and depth of the impact (24–27). Researchers used immature pigs to establish CCI WD models to mimic TBI damage in human children (28). The reason why piglets were chosen is that, unlike other animals, pig brain growth peaks around birth and changes in myelination and water content during development are similar to those in human brain development. After CCI, 1-month-old piglets exhibited focal pathophysiology, replicating the TBI reactions observed in preschool and early childhood, where brain swelling was the most prominent. The CCI model of piglets has also been used to identify relevant TBI biomarkers in peripheral blood and to experiment with intervention therapies that have been proposed for clinical translation. Compared with the Shohami, Feeney, Shapira and Marmarou WD models, the CCI model improved mechanical factors and significantly reduced the fatality rate. A brain stereo locator can also be used to accurately locate craniocerebral strike, with more accurate force. In addition, after impact, the impact head can be automatically and rapidly removed to avoid damage due to extrusion or secondary damage due to rebound (12, 26, 27). In conclusion, the CCI model is more accurate, reproducible and stable, assisting the study of TBI biomechanics.

### 2.2. Mechanical force-induced TBI cellular models

TBI cellular models involve mechanical transection and cell stretch injury. *In vitro* models of mechanical transection, use nerve cell protrusion on the petri dish separated from the cell body by a fine plastic needle, blade or laser, simulating a puncture wound, a penetrating skull fracture or various brain tissue lesions after TBI. Faden et al. (29) used an impact device with 28 stainless steel blades to induce mechanical damage on cultured rat cortical neuron cells. The cutting device produced uniform incisions in the cell layer of the 96-well tissue culture plate with a spacing of 1.2 mm. After 24 h, cell viability was measured by release of lactate dehydrogenase (LDH). The results showed that the cutting device directly caused cell death under the leaf, and within 24 h, the nerve cells around the wound gradually died. Cengiz et al. (30) cultured adult mouse dorsal root ganglion neurons and transected their elongated nerve fibers with a precise laser beam. The cell preparation was observed continuously with time-lapse microscope for 24 h. The more distal the incisions, the longer the degradation field, and thicker axons degraded faster than thinner axons. The advantage of this model was that the position of cutting and the degree of damage were controlled precisely. The model was then simplified by mechanical cutting of cultured rat cortical neurons using a simpler 200 μl yellow spear (diameter 1.5 mm) (31). This method can be used to establish TBI damage of different degrees according to different scratch areas. This simple model requires no special equipment and is easy to operate and effective. However, there is no strict standard for mechanical damage parameters, and damage is only graded by a number of damaged cells. In cellular models of stretch injury, different degrees of stretch caused altered cell morphology, to study biomechanical effects of TBI. widely used model employs compressed gas to deform a clamped circular plate, deforming attached nerve cells, resulting in mild, moderate or severe damage according to the different pressure (32–34). The disadvantages of this model include: uneven deformation at a high deformation rate, and the necessity to verify cellular adhesion to the substrate. Another widely used model involves a microfluidic device applying gas pressure to a pneumatic channel below a flexible polydimethylsiloxane (PDMS) film, which deforms causing axon tensile damage (35). The advantage of a microfluidic device model is that specific areas of nerve cells (such as cell body or axon) can be specifically damaged. However, its disadvantage is that bulky pneumatic devices are needed, and the equipment and instruments are complicated (36). Currently, stretch injury is considered the gold standard for simulating subfatal traumatic neuronal injury. Its advantages include standardized and reproducible damage, as well as direct, real-time measurement of all biomechanical aspects. It is the most widely used *in vitro* TBI model (37–39).

## 3. Pressure injury TBI models

TBI animal models of pressure injury largely include Fluid percussion injury (FPI) and Penetrating encephaliclide-like brain injury (PBBI). FPI causes brain tissue deformation and displacement through rapid injection of a certain amount of normal saline into the cranial cavity, resulting in brain injury. The severity of the resulting injury depends on the intensity of the pressure pulse. FPI can replicate human pathophysiological features such as intracranial hemorrhage, brain swelling and progressive gray matter damage after TBI. It is mainly used to replicate clinical TBI without skull fracture (40–46). The FPI model can be divided into central (sutures above sagittal), parsagittal (distance from midline <3.5 mm) and lateral (from the center line >3.5 mm; LFPI) model depending on the location of skull penetration. Early FPI models largely controlled damage severity by controlling the single variable of height of pendulum fall. In order to improve repeatability, Kabadi et al. (47) developed a microprocessor controlled pneumatic device enabling precise control of impact pressure and residence time to reduce the differences between tests. Cognitive dysfunction and neurobehavioral disorders generated by LFPI models are common clinical symptoms in TBI patients. However, due to brain stem damage and prolonged apnea, FPI models have a higher mortality rate than other models. The selection of craniotomy site in rat LFPI models is very important to the degree of injury. Therefore, the location of craniotomy should be precisely controlled to improve the reliability and repeatability of the model. The PBBI model simulates increased intracranial pressure with impact by a high-powered probe with a shock wave that creates a temporary chamber in the brain many times the size of the projectile itself. The degree of damage depends on the ejection path and the energy transferred. Several new PBBI rodent models have been developed. Davis et al. (48) projected the PBBI probe into the right hemisphere of the brain, through the bone window, to a depth of 1.2 cm. The elastic head of the probe was rapidly filled with water and expanded using a computer program, resulting in an oval balloon with a volume equal to 10% of the brain volume. These PBBI models caused white and gray matter injury, brain edema, epilepsy, cortical diffusion, glial cell proliferation, neuroinflammation and so on. and resulting sensory impairment and cognitive dysfunction. Compared with other TBI models, PBBI can cause extensive intracerebral hemorrhage in the entire primary lesion due to injury and the temporary lumen formed (49). PBBI model are the mechanisms of moderate to severe craniocerebral injury.

Pressure injury models include Compression and Vacuum assisted injury models. The pressure injury model in nerve cells replicates the closed brain injury or FPI model by applying pressure to cultured cells to cause damage. However, in order to get a cellular response, pressure must be increased far beyond the levels that occur during TBI. Under these hydrostatic pressure conditions, brain deformation is likely to be minor because brain tissue is almost incompressible and therefore much higher pressures (around 15 atmospheres) are required to cause damage. The researchers cultured nerve cells, connected to a sealed pressurizer, injecting a nitrogen and oxygen mixture, to give different levels of pressure. Cell volume increased after pressure, with edema became more obvious the higher the pressure. The pressure injury model simulates the clinical pathophysiology after TBI, and the method is simple, the conditions are easy to control, with the degree of injury titrated by adjusting the pressure value. It can be applied to the study of mechanical damage to nerve cells in the CNS, as well as to the study of secondary damage after TBI (50, 51). Negative pressure drainage nerve cell models damage axons by using microfluidic devices and laboratory vacuum. Once axonal growth reaches an adjacent compartment, brief vacuuming of the second compartment with a Pasteur suction tube by creates a bubble, shearing only the axon in the second compartment, causing axon damage without affecting the cell body, which can then be used to screen potential treatments for axon regeneration. Microfluidics and vacuum based damage mechanisms can also be used to simulate and characterize acute axonal degeneration (AAD) (36). Zhou et al. (52) used a microfluidic vacuum inhalation injury model to examine the pathway leading to the observed reduced regeneration of mature axons after injury. In mature axons, the anabolic enzyme (SNPH) -mediated mitochondrial anchoring hinders mitochondrial transport, resulting in energy defects at the damaged site. Enhanced mitochondrial transport by deletion of the SNPH gene promotes axon regeneration after injury by increasing mitochondrial transport and maintaining ATP supply to damaged axons. Therefore, the vacuum inhalation injury model can characterize mitochondrial transport and energy supply of damaged axons, providing a new therapeutic strategy for axon regeneration (36). The disadvantage of the vacuum suction method is the high fluid resistance required between interconnected compartments to limit damage to specific neuronal areas. This resistance is usually provided by a microgroove in the microfluidic device allowing the duration and strength of the vacuum to be carefully adjusted to minimize damage to non-specific areas.

## 4. Blast-induced injury (BTBI) models

Craniocerebral blast injury is mainly caused by blast wave and projectiles; the main type of injury in modern warfare. Domestic and foreign scholars have established various models of BTBI. Among the most common are the free field explosion model, the Blast tube model, the small explosion source model, and the Advanced Blast Simulator; (ABS) (53–56). The ABS model does not use explosives, but rather compressed gas as power. A cylindrical tube is divided into two chambers; the pressurized and the test areas by a thin film of special material. When the pressure in the pressurized area rises to a certain extent, the diaphragm is broken and a shockwave is generated, causing damage to the animal’s head, placed in the test area. Uylissa A. Rodriguez et al. (56) adopted a shock tube test area of 2 m containing the rat’s head, with a compression area 2.54 m in length and a diaphragm thickness of 0.4 mm. An animal model of BTBI was established by pressurizing air to about 1,230 kPA to break the diaphragm causing a shock wave, resulting in head injury in the rat. Blast waves can damage cerebral blood vessels, neurons, glial cells and blood–brain barrier, leading to the activation of microglia and neuroinflammatory response (57, 58). Like other injury models, bTBI also exhibits pathological results such as ventricular enlargement, gray/white matter atrophy, axonal injury, cell apoptosis, and neuroinflammation (58, 59). The mechanism of brain damage caused by shock waves is currently unclear.

In the same way, cultured nerve cells and brain tissue sections were placed in the test area of the shock tube to establish a BTBI model *in vitro*. Campos-Pires et al. (60) oriented the cells of the mouse hippocampal brain slice toward the shock tube, and established a trauma model with different impact pressures. The degree of damage degree increased with the increase in impact pressure peak and shock wave. The main mode of cell death induced by the shock wave was apoptosis. Researchers found that after cells were damaged by shock waves, the levels of adenosine triphosphate (ATP) decreased, while LDH and reactive oxygen species were upregulated (61, 62). Ravin et al. (63) used a pneumatic device based on an air gun to simulate the blast shock wave, and established a BTBI cell model by using primary cultured rat CNS cortical tissue and human CNS cortical tissue. They found that the expression of reactive astrocyte markers GFAP and protease matrix metallopeptidase 9 in human central nervous system cells significantly increased 24 h after shock wave stimulation. Interestingly, they found that human astrocyte were more responsive to injury than rat derived astrocyte. The ABS model has its own important shortcomings. First, the physical characteristics of the gas-driven shock wave may be different from that of the explosion shock wave; second, the diaphragm fragment may impact the subjects; third, the efflux effect generated near the tube outlet may have impacted the subjects (53). The main advantages of the ABS model are its high safety, indoor operation, and greatly reduced external interference. Shock waves of different sizes can be generated by adjusting the material of the diaphragm (64–66). The ABS model is the most widely used model in BTBI research.

## 5. Repeated minor trauma injury models (r-mTBI)

R-mTBI usually occurs in contact sports (eg. boxing, basketball, football, rugby) and in domestic violence (12, 67). There is growing evidence that repeated concussions can lead to behavioral abnormalities and pathological changes, and several models of r-mTBI have been established. These include the CCI, WD, FPI Blast-TBI, and Cell stretch injury models (68–72). R-mTBI over a short period of time can cause diffuse axonal injury and chronic neuroinflammation. These pathophysiological phenomena are closely related to neurodegenerative diseases such as Alzheimer’s disease and Parkinson’s syndrome. The microtubule associated protein tau is an indispensable part of the pathogenesis of Alzheimer’s disease (AD) and several related diseases called tau disease, where tau is deposited in the affected brain regions. Research has shown that changes in soluble tau proteins, including phosphorylation, are involved in inducing neuronal death. Therefore, the method of reducing tau phosphorylation may exert the neuroprotective effect of neurodegenerative diseases such as AD by reducing the generation of amyloid protein (73). Research has shown that tau phosphorylation may be regulated by metal ions such as iron, zinc, and copper, which are themselves associated with neurodegenerative diseases such as aging and Alzheimer’s disease (74). The results of most studies show that r-mTBI will lead to pathological tau formation, metal homeostasis disorder, tau hyperphosphorylation, astrocyte proliferation, microglia proliferation and brain atrophy, as well as progressive learning and cognitive disorders that continue to develop for a long time after the injury stops. Of course, it is important to emphasize that not all r-mTBI studies have reported tau pathology, which may be because TBI models There are differences in experimental design parameters, species, animal age, and detected time points (74). Although mild brain damage is often overlooked, with the intensification of population aging, r-mTBI will be increasingly studied by researchers. Therefore, developing sufficient r-mTBI models and considering more factors such as species, gender, age, acute phase, subacute phase, and chronic phase, provides more sufficient evidence on how r-mTBI leads to pathological tau formation, metal homeostasis disorders, and motor and cognitive deficits, thereby jointly leading to neurodegenerative diseases.

## 6. Conclusions and future directions

Although the application of TBI models in the study of brain injury has made some progress, there are still some insuperable shortcomings (Table 1). The brains of commonly used TBI model animals (especially rodents) are physiologically similar to human brains, to a certain extent, but there are still significant differences in brain structure and function, such as brain geometry, cranial Angle, cyclotron complexity, and gray to white matter ash ratio (12, 41). Many TBI model studies strictly did not measure physiological variables before and after TBI, including CO2, and O2 partial pressures, pH, blood pressure and brain temperature, etc. These variables are important in determining the body’s pathophysiological response to injury and treatment. In addition, age, sex and species have an impact on TBI results (3, 4, 76, 77, 79, 80), and more research is needed. The limitation of *in vitro* TBI models is that tissue cells may produce harmful stress responses *in vitro*. Secondly, tissue cells may have been damaged in the process of sampling, which may influence experimental tissue damage to a certain extent. *In vitro* TBI models should focus on reducing the influence of extracellular environment (such as blood, activated macrophages, etc.) on nerve cells and reducing the damage caused by tissue cells in the process of sampling (75, 78, 81–83). Sometimes studies based on *in vitro* and *in vivo* models produce conflicting results, but this does not mean that *in vitro* models are inaccurate and may relate to environmental differences (e.g., inflammatory responses, temperature regulation, oxygenation, or local ion concentrations) (12, 54, 82, 84–86). Both types of TBI models have advantages and disadvantages. Therefore, different types of *in vitro* and *in vivo* TBI models should be combined, when studying a new treatment or drug, to simulate different pathobiological reactions caused during injury. Cross-validation in this way can make the experimental results more robust and reliable, and reduce the false positive neuroprotective effect of some drugs or treatments.

**Table 1:** Characteristics of the commonly used TBI animal and cell models.

Types of TBI models			Types of injury	Limitations	Advantages
Mechanical injury models	Animal models	Feeney (16–20, 75)	Contusion cerebral cortex; Cerebral concussion	A bone window is required High fatality rate	Simple methods; Easy control
		Shohami (21–24)	Induced mixed; Diffuse injury	High fatality rate; Poor repeatability	Simple methods; Low cost
		Marmarou (10, 14, 15, 21)	Diffuse injury; Axonal injury	High fatality rate	Simple methods; Easy control
		CCI (24, 25, 30–32, 68)	Cortical loss; Cerebral concussion	Expensive equipment; A bone window is required	Good repeatability Accuracy of injury
	Cell models	Transection (29–31)	Axonal damage and puncture wounds	Need for standardization	No special equipment and instrument conditions are required
		Stretch (32–39, 76–78)	Axonal damage to nerve cells	Complex instrument; Expensive equipment	Precise damage to specific areas of the cell
Pressure injury models	Animal models	FPI (40–47, 70)	Intracranial hemorrhage and brain swelling	The mechanism of injury is different from the clinical situation	Good repeatability; High stability
		PBBI (48, 49)	Intracranial hemorrhage and increased intracranial pressure	Standardization and special equipment are required	The injury was similar to the clinical one
	Cell models	Compression (50, 51)	Increased intracranial pressure was replicated	Pressure control device is required	Simple methods; Easy control of conditions
		Vacuum helper cell damage (36, 52)	Axonal damage to nerve cells	Pressure control needs to be refined	Accuracy of injury; Simple methods
Blast injury models	Animal models	BTBI model (54–66)	Blast shock wave damage	Special equipment; Jet flow effect	The injuries were similar to war wounds
	Cell models	ABS cell model (60, 61, 63, 66)	Blast shock wave damage	Diaphragm fragment shadow	Indoor operation; High safety
R-mTBI (12, 67–72, 74)			Diffuse brain injury	Need for standardization	The injury was similar to the clinical one

## Author contributions

QZ and FX designed the overall project. FX, HoL, and QZ analyzed the data and wrote the manuscript. HuL, and JZ revised the manuscript. All authors contributed to the article and approved the submitted version.

## Funding

This work was supported by the Key Scientific Research Project in Colleges and Universities of Henan Province (grant number: 22A310002) and National Natural Science Foundation of China (grant number: 31500828).

## Conflict of interest

The authors declare that the research was conducted in the absence of any commercial or financial relationships that could be construed as a potential conflict of interest.

## Publisher’s note

All claims expressed in this article are solely those of the authors and do not necessarily represent those of their affiliated organizations, or those of the publisher, the editors and the reviewers. Any product that may be evaluated in this article, or claim that may be made by its manufacturer, is not guaranteed or endorsed by the publisher.

## References

[1] Ruff RL Riechers RG. Effective treatment of traumatic brain injury: learning from experience. JAMA. (2012) 308:2032–3. doi: 10.1001/jama.2012.14008 23168827

[2] Maas AIR Menon DK Manley GT Abrams M Åkerlund C Andelic N. Traumatic brain injury: progress and challenges in prevention, clinical care, and research. Lancet Neurol. (2022) 21:1004–60. doi: 10.1016/S1474-4422(22)00309-X, PMID: 36183712 PMC10427240

[3] Taylor CA Bell JM Breiding MJ Xu L. Traumatic brain injury-related emergency department visits, hospitalizations, and deaths-United States, 2007 and 2013. MMWR Surveill Summ. (2017) 66:1–16. doi: 10.15585/mmwr.ss6609a1, PMID: 28301451 PMC5829835

[4] Majdan M Plancikova D Brazinova A Rusnak M Nieboer D Feigin V. Epidemiology of traumatic brain injuries in Europe: a cross-sectional analysis. Lancet Public Health. (2016) 1:e76–83. doi: 10.1016/S2468-2667(16)30017-2 29253420

[5] Jiang JY Gao GY Feng JF Mao Q Chen LG Yang XF. Traumatic brain injury in China. Lancet Neurol. (2019) 18:286–95. doi: 10.1016/S1474-4422(18)30469-1 30784557

[6] Hicks AJ Ponsford JL Spitz G Dore V Krishnadas N Roberts C. β-Amyloid and tau imaging in chronic traumatic brain injury: a cross-sectional study. Neurology. (2022) 99:e1131–41. doi: 10.1212/WNL.0000000000200857, PMID: 36096678

[7] VanItallie TB. Traumatic brain injury (TBI) in collision sports: possible mechanisms of transformation into chronic traumatic encephalopathy (CTE). Metab Clin Exp. (2019) 100:153943. doi: 10.1016/j.metabol.2019.07.007, PMID: 31610856

[8] Golub VM Reddy DS. Post-traumatic epilepsy and comorbidities: advanced models, molecular mechanisms, biomarkers, and novel therapeutic interventions. Pharmacol Rev. (2022) 74:387–438. doi: 10.1124/pharmrev.121.000375, PMID: 35302046 PMC8973512

[9] Shi K Zhang J Dong JF Shi FD. Dissemination of brain inflammation in traumatic brain injury. Cell Mol Immunol. (2019) 16:523–30. doi: 10.1038/s41423-019-0213-5, PMID: 30846842 PMC6804599

[10] Ebrahimi H Kazem Nezhad S Farmoudeh A Babaei A Ebrahimnejad P Akbari E. Design and optimization of metformin-loaded solid lipid nanoparticles for neuroprotective effects in a rat model of diffuse traumatic brain injury: a biochemical, behavioral, and histological study. Eur J Pharmaceut Biopharmaceut Off J Arbeitsgemeinschaft Pharmazeutische Verfahrenstechnik eV. (2022) 181:122–35. doi: 10.1016/j.ejpb.2022.10.018, PMID: 36307002

[11] Feeney DM Boyeson MG Linn RT Murray HM Dail WG. Responses to cortical injury: I. methodology and local effects of contusions in the rat. Brain Res. (1981) 211:67–77. doi: 10.1016/0006-8993(81)90067-6, PMID: 7225844

[12] Xiong Y Mahmood A Chopp M. Animal models of traumatic brain injury. Nat Rev Neurosci. (2013) 14:128–42. doi: 10.1038/nrn3407, PMID: 23329160 PMC3951995

[13] Khellaf A Khan DZ Helmy A. Recent advances in traumatic brain injury. J Neurol. (2019) 266:2878–89. doi: 10.1007/s00415-019-09541-4, PMID: 31563989 PMC6803592

[14] Marmarou A Foda MA van den Brink W Campbell J Kita H Demetriadou K. A new model of diffuse brain injury in rats. Part I: pathophysiology and biomechanics. J Neurosurg. (1994) 80:291–300. doi: 10.3171/jns.1994.80.2.0291 8283269

[15] Chakraborty N Hammamieh R Gautam A Miller SA Condlin ML Jett M. TBI weight-drop model with variable impact heights differentially perturbs hippocampus-cerebellum specific transcriptomic profile. Exp Neurol. (2021) 335:113516. doi: 10.1016/j.expneurol.2020.113516, PMID: 33172833

[16] Pang AL Xiong LL Xia QJ Liu F Wang YC Liu F. Neural stem cell transplantation is associated with inhibition of apoptosis, Bcl-xL Upregulation, and recovery of neurological function in a rat model of traumatic brain injury. Cell Transplant. (2017) 26:1262–75. doi: 10.1177/0963689717715168, PMID: 28933221 PMC5657736

[17] Chen X Wu S Chen C Xie B Fang Z Hu W. Omega-3 polyunsaturated fatty acid supplementation attenuates microglial-induced inflammation by inhibiting the HMGB1/TLR4/NF-κB pathway following experimental traumatic brain injury. J Neuroinflammation. (2017) 14:143. doi: 10.1186/s12974-017-0917-3, PMID: 28738820 PMC5525354

[18] Jia J Chen F Wu Y. Recombinant PEP-1-SOD1 improves functional recovery after neural stem cell transplantation in rats with traumatic brain injury. Exp Ther Med. (2018) 15:2929–35. doi: 10.3892/etm.2018.5781, PMID: 29599832 PMC5867477

[19] He H Liu W Zhou Y Liu Y Weng P Li Y. Sevoflurane post-conditioning attenuates traumatic brain injury-induced neuronal apoptosis by promoting autophagy via the PI3K/AKT signaling pathway. Drug Des Devel Ther. (2018) 12:629–38. doi: 10.2147/DDDT.S158313, PMID: 29606856 PMC5868589

[20] Wang Z Li J Wang A Wang Z Wang J Yuan J. Sevoflurane inhibits traumatic brain injury-induced neuron apoptosis via EZH2-Downregulated KLF4/p38 Axis. Front Cell Dev Biol. (2021) 9:658720. doi: 10.3389/fcell.2021.658720, PMID: 34422795 PMC8371463

[21] Bodnar CN Roberts KN Higgins EK Bachstetter AD. A systematic review of closed Head injury models of mild traumatic brain injury in mice and rats. J Neurotrauma. (2019) 36:1683–706. doi: 10.1089/neu.2018.6127, PMID: 30661454 PMC6555186

[22] Shapira Y Shohami E Sidi A Soffer D Freeman S Cotev S. Experimental closed head injury in rats: mechanical, pathophysiologic, and neurologic properties. Crit Care Med. (1988) 16:258–65. doi: 10.1097/00003246-198803000-00010 3277783

[23] Flierl MA Stahel PF Beauchamp KM Morgan SJ Smith WR Shohami E. Mouse closed head injury model induced by a weight-drop device. Nat Protoc. (2009) 4:1328–37. doi: 10.1038/nprot.2009.148, PMID: 19713954

[24] Ma X Aravind A Pfister BJ Chandra N Haorah J. Animal models of traumatic brain injury and assessment of injury severity. Mol Neurobiol. (2019) 56:5332–45. doi: 10.1007/s12035-018-1454-5 30603958

[25] Lighthall JW. Controlled cortical impact: a new experimental brain injury model. J Neurotrauma. (1988) 5:1–15. doi: 10.1089/neu.1988.5.1 3193461

[26] Zhao QH Xie F Guo DZ Ju FD He J Yao TT. Hydrogen inhalation inhibits microglia activation and neuroinflammation in a rat model of traumatic brain injury. Brain Res. (2020) 1748:147053. doi: 10.1016/j.brainres.2020.147053, PMID: 32814064

[27] Hu J Wang X Chen X Fang Y Chen K Peng W. Hydroxychloroquine attenuates neuroinflammation following traumatic brain injury by regulating the TLR4/NF-κB signaling pathway. J Neuroinflammation. (2022) 19:71. doi: 10.1186/s12974-022-02430-0 35346242 PMC8961949

[28] Shultz SR McDonald SJ Vonder Haar C Meconi A Vink R van Donkelaar P. The potential for animal models to provide insight into mild traumatic brain injury: translational challenges and strategies. Neurosci Biobehav Rev. (2017) 76:396–414. doi: 10.1016/j.neubiorev.2016.09.014, PMID: 27659125

[29] Faden AI Movsesyan VA Knoblach SM Ahmed F Cernak I. Neuroprotective effects of novel small peptides *in vitro* and after brain injury. Neuropharmacology. (2005) 49:410–24. doi: 10.1016/j.neuropharm.2005.04.001, PMID: 15907950

[30] Cengiz N Oztürk G Erdoğan E Him A Oğuz EK. Consequences of neurite transection *in vitro*. J Neurotrauma. (2012) 29:2465–74. doi: 10.1089/neu.2009.0947, PMID: 20121423 PMC3471124

[31] Liu W Chen Y Meng J Wu M Bi F Chang C. Ablation of caspase-1 protects against TBI-induced pyroptosis *in vitro* and *in vivo*. J Neuroinflammation. (2018) 15:48. doi: 10.1186/s12974-018-1083-y, PMID: 29458437 PMC5817788

[32] Saykally JN Hatic H Keeley KL Jain SC Ravindranath V Citron BA. Withania somnifera extract protects model neurons from *in vitro* traumatic injury. Cell Transplant. (2017) 26:1193–201. doi: 10.1177/0963689717714320, PMID: 28933215 PMC5657733

[33] Cater HL Sundstrom LE Morrison B. Temporal development of hippocampal cell death is dependent on tissue strain but not strain rate. J Biomech. (2006) 39:2810–8. doi: 10.1016/j.jbiomech.2005.09.023 16289515

[34] Salvador E Burek M Förster CY. Stretch and/or oxygen glucose deprivation (OGD) in an *in vitro* traumatic brain injury (TBI) model induces calcium alteration and inflammatory cascade. Front Cell Neurosci. (2015) 9:323. doi: 10.3389/fncel.2015.00323, PMID: 26347611 PMC4543908

[35] Yap YC King AE Guijt RM Jiang T Blizzard CA Breadmore MC. Mild and repetitive very mild axonal stretch injury triggers cystoskeletal mislocalization and growth cone collapse. PLoS One. (2017) 12:e0176997. doi: 10.1371/journal.pone.0176997, PMID: 28472086 PMC5417565

[36] Shrirao AB Kung FH Omelchenko A Schloss RS Boustany NN Zahn JD. Microfluidic platforms for the study of neuronal injury *in vitro*. Biotechnol Bioeng. (2018) 115:815–30. doi: 10.1002/bit.26519, PMID: 29251352 PMC5831486

[37] Kumaria A. *In vitro* models as a platform to investigate traumatic brain injury. Alternat Lab Anim ATLA. (2017) 45:201–11. doi: 10.1177/026119291704500405, PMID: 28994300

[38] Chaves RS Tran M Holder AR Balcer AM Dickey AM Roberts EA. Amyloidogenic processing of amyloid precursor protein drives stretch-induced disruption of axonal transport in hiPSC-derived neurons. J Neurosci Off J Soc Neurosci. (2021) 41:10034–53. doi: 10.1523/JNEUROSCI.2553-20.2021, PMID: 34663629 PMC8660048

[39] Wu YH Rosset S Lee TR Dragunow M Park T Shim V. *In vitro* models of traumatic brain injury: a systematic review. J Neurotrauma. (2021) 38:2336–72. doi: 10.1089/neu.2020.7402, PMID: 33563092

[40] Dixon CE Lyeth BG Povlishock JT Findling RL Hamm RJ Marmarou A. A fluid percussion model of experimental brain injury in the rat. J Neurosurg. (1987) 67:110–9. doi: 10.3171/jns.1987.67.1.0110 3598659

[41] Morales DM Marklund N Lebold D Thompson HJ Pitkanen A Maxwell WL. Experimental models of traumatic brain injury: do we really need to build a better mousetrap? Neuroscience. (2005) 136:971–89. doi: 10.1016/j.neuroscience.2005.08.030, PMID: 16242846

[42] Alder J Fujioka W Lifshitz J Crockett DP Thakker-Varia S. Lateral fluid percussion: model of traumatic brain injury in mice. J Visual Exp JoVE. (2011) 54:3063. doi: 10.3791/3063, PMID: 21876530 PMC3217637

[43] Liu YR Cardamone L Hogan RE Gregoire MC Williams JP Hicks RJ. Progressive metabolic and structural cerebral perturbations after traumatic brain injury: an *in vivo* imaging study in the rat. J Nucl Med Off Publ Soc Nucl Med. (2010) 51:1788–95. doi: 10.2967/jnumed.110.078626 21051651

[44] Evans LP Newell EA Mahajan M Tsang SH Ferguson PJ Mahoney J. Acute vitreoretinal trauma and inflammation after traumatic brain injury in mice. Ann Clin Transl Neurol. (2018) 5:240–51. doi: 10.1002/acn3.523, PMID: 29560370 PMC5846452

[45] Song H Chen C Kelley B Tomasevich A Lee H Dolle JP. Traumatic brain injury recapitulates developmental changes of axons. Prog Neurobiol. (2022) 217:102332. doi: 10.1016/j.pneurobio.2022.102332, PMID: 35870679 PMC9454890

[46] Wangler LM Bray CE Packer JM Tapp ZM Davis AC O'Neil SM. Amplified gliosis and interferon-associated inflammation in the aging brain following diffuse traumatic brain injury. J Neurosci Off J Soc Neurosci. (2022) 42:9082–96. doi: 10.1523/JNEUROSCI.1377-22.2022, PMID: 36257689 PMC9732830

[47] Kabadi SV Hilton GD Stoica BA Zapple DN Faden AI. Fluid-percussion-induced traumatic brain injury model in rats. Nat Protoc. (2010) 5:1552–63. doi: 10.1038/nprot.2010.112, PMID: 20725070 PMC3753081

[48] Davis AR Shear DA Chen Z Lu XC Tortella FC. A comparison of two cognitive test paradigms in a penetrating brain injury model. J Neurosci Methods. (2010) 189:84–7. doi: 10.1016/j.jneumeth.2010.03.012, PMID: 20346980

[49] Shear DA Lu XC Pedersen R Wei G Chen Z Davis A. Severity profile of penetrating ballistic-like brain injury on neurofunctional outcome, blood-brain barrier permeability, and brain edema formation. J Neurotrauma. (2011) 28:2185–95. doi: 10.1089/neu.2011.1916, PMID: 21644814

[50] Popova D Karlsson J Jacobsson SOP. Comparison of neurons derived from mouse P19, rat PC12 and human SH-SY5Y cells in the assessment of chemical- and toxin-induced neurotoxicity. BMC Pharmacol Toxicol. (2017) 18:42. doi: 10.1186/s40360-017-0151-8, PMID: 28583171 PMC5460426

[51] Smith ME Eskandari R. A novel technology to model pressure-induced cellular injuries in the brain. J Neurosci Methods. (2018) 293:247–53. doi: 10.1016/j.jneumeth.2017.10.004, PMID: 28993205

[52] Zhou B Yu P Lin MY Sun T Chen Y Sheng ZH. Facilitation of axon regeneration by enhancing mitochondrial transport and rescuing energy deficits. J Cell Biol. (2016) 214:103–19. doi: 10.1083/jcb.201605101, PMID: 27268498 PMC4932375

[53] Kovacs SK Leonessa F Ling GS. Blast TBI models, neuropathology, and implications for seizure risk. Front Neurol. (2014) 5:47. doi: 10.3389/fneur.2014.00047, PMID: 24782820 PMC3988378

[54] Risling M Plantman S Angeria M Rostami E Bellander BM Kirkegaard M. Mechanisms of blast induced brain injuries, experimental studies in rats. Neuro Image. (2011) 54:S89–97. doi: 10.1016/j.neuroimage.2010.05.031, PMID: 20493951

[55] Aravind A Ravula AR Chandra N Pfister BJ. Behavioral deficits in animal models of blast traumatic brain injury. Front Neurol. (2020) 11:990. doi: 10.3389/fneur.2020.00990, PMID: 33013653 PMC7500138

[56] Rodriguez UA Zeng Y Deyo D Parsley MA Hawkins BE Prough DS. Effects of mild blast traumatic brain injury on cerebral vascular, Histopathological, and behavioral outcomes in rats. J Neurotrauma. (2018) 35:375–92. doi: 10.1089/neu.2017.5256, PMID: 29160141 PMC5784797

[57] Kobeissy F Mondello S Tümer N Toklu HZ Whidden MA Kirichenko N. Assessing neuro-systemic & behavioral components in the pathophysiology of blast-related brain injury. Front Neurol. (2013) 4:186. doi: 10.3389/fneur.2013.00186, PMID: 24312074 PMC3836009

[58] Gama Sosa MA De Gasperi R Perez Garcia GS Sosa H Searcy C Vargas D. Lack of chronic neuroinflammation in the absence of focal hemorrhage in a rat model of low-energy blast-induced TBI. Acta Neuropathol Commun. (2017) 5:80. doi: 10.1186/s40478-017-0483-z, PMID: 29126430 PMC6389215

[59] Tchantchou F Fourney WL Leiste UH Vaughan J Rangghran P Puche A. Neuropathology and neurobehavioral alterations in a rat model of traumatic brain injury to occupants of vehicles targeted by underbody blasts. Exp Neurol. (2017) 289:9–20. doi: 10.1016/j.expneurol.2016.12.001, PMID: 27923561

[60] Campos-Pires R Koziakova M Yonis A Pau A Macdonald W Harris K. Xenon protects against blast-induced traumatic brain injury in an *in vitro* model. J Neurotrauma. (2018) 35:1037–44. doi: 10.1089/neu.2017.5360, PMID: 29285980 PMC5899289

[61] Arun P Spadaro J John J Gharavi RB Bentley TB Nambiar MP. Studies on blast traumatic brain injury using *in-vitro* model with shock tube. Neuroreport. (2011) 22:379–84. doi: 10.1097/WNR.0b013e328346b138, PMID: 21532394

[62] Vogel EW 3rd Morales FN Meaney DF Bass CR Morrison B 3rd. Phosphodiesterase-4 inhibition restored hippocampal long term potentiation after primary blast. Exp Neurol. (2017) 293:91–100. doi: 10.1016/j.expneurol.2017.03.025 28366471 PMC6016024

[63] Ravin R Blank PS Busse B Ravin N Vira S Bezrukov L. Blast shockwaves propagate Ca(2+) activity via purinergic astrocyte networks in human central nervous system cells. Sci Rep. (2016) 6:25713. doi: 10.1038/srep25713, PMID: 27162174 PMC4861979

[64] Rosenfeld JV McFarlane AC Bragge P Armonda RA Grimes JB Ling GS. Blast-related traumatic brain injury. Lancet Neurol. (2013) 12:882–93. doi: 10.1016/S1474-4422(13)70161-3 23884075

[65] Snapper DM Reginauld B Liaudanskaya V Fitzpatrick V Kim Y Georgakoudi I. Development of a novel bioengineered 3D brain-like tissue for studying primary blast-induced traumatic brain injury. J Neurosci Res. (2023) 101:3–19. doi: 10.1002/jnr.25123, PMID: 36200530

[66] Campos-Pires R Yonis A Macdonald W Harris K Edge CJ Mahoney PF. A novel *in vitro* model of blast traumatic brain injury. J Vis Exp JoVE. (2018). doi: 10.3791/58400 30614488

[67] Pham L Wright DK O'Brien WT Bain J Huang C Sun M. Behavioral, axonal, and proteomic alterations following repeated mild traumatic brain injury: novel insights using a clinically relevant rat model. Neurobiol Dis. (2021) 148:105151. doi: 10.1016/j.nbd.2020.105151, PMID: 33127468

[68] Yu F Shukla DK Armstrong RC Marion CM Radomski KL Selwyn RG. Repetitive model of mild traumatic brain injury produces cortical abnormalities detectable by magnetic resonance diffusion imaging, histopathology, and behavior. J Neurotrauma. (2017) 34:1364–81. doi: 10.1089/neu.2016.4569, PMID: 27784203 PMC5385606

[69] Kane MJ Angoa-Pérez M Briggs DI Viano DC Kreipke CW Kuhn DM. A mouse model of human repetitive mild traumatic brain injury. J Neurosci Methods. (2012) 203:41–9. doi: 10.1016/j.jneumeth.2011.09.003 21930157 PMC3221913

[70] Shultz SR MacFabe DF Foley KA Taylor R Cain DP. Sub-concussive brain injury in the long-Evans rat induces acute neuroinflammation in the absence of behavioral impairments. Behav Brain Res. (2012) 229:145–52. doi: 10.1016/j.bbr.2011.12.015, PMID: 22245525

[71] Skotak M Wang F Chandra N. An *in vitro* injury model for SH-SY5Y neuroblastoma cells: effect of strain and strain rate. J Neurosci Methods. (2012) 205:159–68. doi: 10.1016/j.jneumeth.2012.01.001, PMID: 22257521

[72] Ondek K Brevnova O Jimenez-Ornelas C Vergara A Zwienenberg M Gurkoff G. A new model of repeat mTBI in adolescent rats. Exp Neurol. (2020) 331:113360. doi: 10.1016/j.expneurol.2020.113360, PMID: 32442552

[73] Hanger DP Anderton BH Noble W. Tau phosphorylation: the therapeutic challenge for neurodegenerative disease. Trends Mol Med. (2009) 15:112–9. doi: 10.1016/j.molmed.2009.01.003 19246243

[74] Juan SMA Daglas M Adlard PA. Tau pathology, metal Dyshomeostasis and repetitive mild traumatic brain injury: an unexplored link paving the way for Neurodegeneration. J Neurotrauma. (2022) 39:902–22. doi: 10.1089/neu.2021.0241, PMID: 35293225

[75] Adamchik Y Frantseva MV Weisspapir M Carlen PL Perez Velazquez JL. Methods to induce primary and secondary traumatic damage in organotypic hippocampal slice cultures. Brain Res Brain Res Protoc. (2000) 5:153–8. doi: 10.1016/s1385-299x(00)00007-6, PMID: 10775835

[76] Puntambekar SS Saber M Lamb BT Kokiko-Cochran ON. Cellular players that shape evolving pathology and neurodegeneration following traumatic brain injury. Brain Behav Immun. (2018) 71:9–17. doi: 10.1016/j.bbi.2018.03.033, PMID: 29601944

[77] Prexl O Bruckbauer M Voelckel W Grottke O Ponschab M Maegele M. The impact of direct oral anticoagulants in traumatic brain injury patients greater than 60-years-old. Scand J Trauma Resusc Emerg Med. (2018) 26:20. doi: 10.1186/s13049-018-0487-0, PMID: 29580268 PMC5870487

[78] Morrison B 3rd Cater HL Benham CD Sundstrom LE. An *in vitro* model of traumatic brain injury utilising two-dimensional stretch of organotypic hippocampal slice cultures. J Neurosci Methods. (2006) 150:192–201. doi: 10.1016/j.jneumeth.2005.06.014, PMID: 16098599

[79] Ercole A Magnoni S Vegliante G Pastorelli R Surmacki J Bohndiek SE. Current and emerging Technologies for Probing Molecular Signatures of traumatic brain injury. Front Neurol. (2017) 8:450. doi: 10.3389/fneur.2017.00450, PMID: 28912750 PMC5582086

[80] Shah EJ Gurdziel K Ruden DM. Mammalian models of traumatic brain injury and a place for Drosophila in TBI research. Front Neurosci. (2019) 13:409. doi: 10.3389/fnins.2019.00409, PMID: 31105519 PMC6499071

[81] Vink R. Large animal models of traumatic brain injury. J Neurosci Res. (2018) 96:527–35. doi: 10.1002/jnr.24079 28500771

[82] Morrison B 3rd Elkin BS Dollé JP Yarmush ML. *In vitro* models of traumatic brain injury. Annu Rev Biomed Eng. (2011) 13:91–126. doi: 10.1146/annurev-bioeng-071910-124706 21529164

[83] Liu N Li Y Jiang Y Shi S Niamnud A Vodovoz SJ. Establishment and application of a novel *in vitro* model of microglial activation in traumatic brain injury. J Neurosci Off J Soc Neurosci. (2022) 43:319–32. doi: 10.1523/JNEUROSCI.1539-22.2022, PMID: 36446585 PMC9838700

[84] Elkin BS Morrison B. Region-specific tolerance criteria for the living brain. Stapp Car Crash J. (2007) 51:127–38. doi: 10.4271/2007-22-0005, PMID: 18278594

[85] Thelin EP Hall CE Gupta K Carpenter KLH Chandran S Hutchinson PJ. Elucidating pro-inflammatory cytokine responses after traumatic brain injury in a human stem cell model. J Neurotrauma. (2018) 35:341–52. doi: 10.1089/neu.2017.5155, PMID: 28978285 PMC5784793

[86] Estrada-Rojo F Martínez-Tapia RJ Estrada-Bernal F Martínez-Vargas M Perez-Arredondo A Flores-Avalos L. Models used in the study of traumatic brain injury. Rev Neurosci. (2018) 29:139–49. doi: 10.1515/revneuro-2017-0028, PMID: 28888093

